# Prognostic value of imaging-detected immune-related adverse events in biliary tract cancer patients receiving durvalumab-based chemotherapy

**DOI:** 10.1007/s00330-026-12645-x

**Published:** 2026-05-28

**Authors:** Jeongin Yoo, Dong Ho Lee, Do-Youn Oh, Jeesun Yoon, Sang Hyub Lee, In Rae Cho, Woo Hyun Paik

**Affiliations:** 1 Department of Radiology, Seoul National University Hospital, Seoul, Korea; 2 Department of Radiology, Seoul National University College of Medicine, Seoul, Korea; 3 Seoul National University Hospital, Cancer Research Institute, Seoul National University College of Medicine, Integrated Major in Innovative Medical Science, Seoul National University Graduate School, Seoul, Korea; 4 Division of Medical Oncology, Department of Internal Medicine, Seoul National University Hospital, Seoul, Korea; 5 Department of Internal Medicine and Liver Research Institute, Seoul National University Hospital, Seoul National University College of Medicine, Seoul, Korea

**Keywords:** Biliary tract neoplasm, Immune checkpoint inhibitor, Drug-related side effects and adverse reactions, Multidetector computed tomography

## Abstract

**Objectives:**

This study aimed to evaluate the incidence and prognostic significance of imaging-detected immune-related adverse events (irAEs) in patients with unresectable biliary tract cancer (BTC) receiving durvalumab combined with gemcitabine and cisplatin.

**Materials and methods:**

This retrospective cohort study included 66 patients (mean age ± standard deviation, 64.7 ± 9.4 years, 35 women) with histologically confirmed unresectable BTC who received a combination of durvalumab, gemcitabine, and cisplatin as the first-line systemic therapy. Clinical records and imaging studies were reviewed to identify irAEs. The primary endpoint was overall survival (OS); secondary endpoints included progression-free survival (PFS), treatment response, and incidence of irAEs. Cox regression analyses were conducted to determine predictors of survival outcomes.

**Results:**

Imaging-detected irAEs were observed in 21.2% (14/66) of patients, with the most frequent findings being bowel wall thickening and transient inflammatory lung nodules. The majority of these events (85.7%) manifested within 90 days of treatment initiation, and only 35.7% (5/14) had clinical symptoms related to irAE. In multivariate analysis, the presence of imaging-detected irAEs was a significant independent predictor of improved OS (hazard ratio (HR) = 0.42; *p* = 0.017) and PFS (HR = 0.43; *p* = 0.016). The median OS was significantly longer in patients with imaging-detected irAEs compared to those without (23.0 vs. 12.0 months; *p* = 0.009).

**Conclusion:**

Imaging-detected irAEs are potent prognostic markers for superior survival in BTC patients receiving durvalumab-based chemotherapy. Given that these events are frequently subclinical and occur early in the treatment course, meticulous radiologic surveillance is essential to identify patients likely to achieve favorable clinical outcomes.

**Key Points:**

***Question*** *The relationship between immune-related adverse events (irAEs) on imaging and treatment outcome in biliary tract cancer patients undergoing durvalumab-based therapy has not been thoroughly investigated.*

***Findings*** *The development of irAEs detected on imaging study was significantly associated with improved overall survival.*

***Clinical relevance*** *Careful imaging evaluation during follow-up to detect early irAE-related change is important, since the occurrence of irAEs may indicate better clinical outcomes.*

**Graphical Abstract:**

**Figure:**
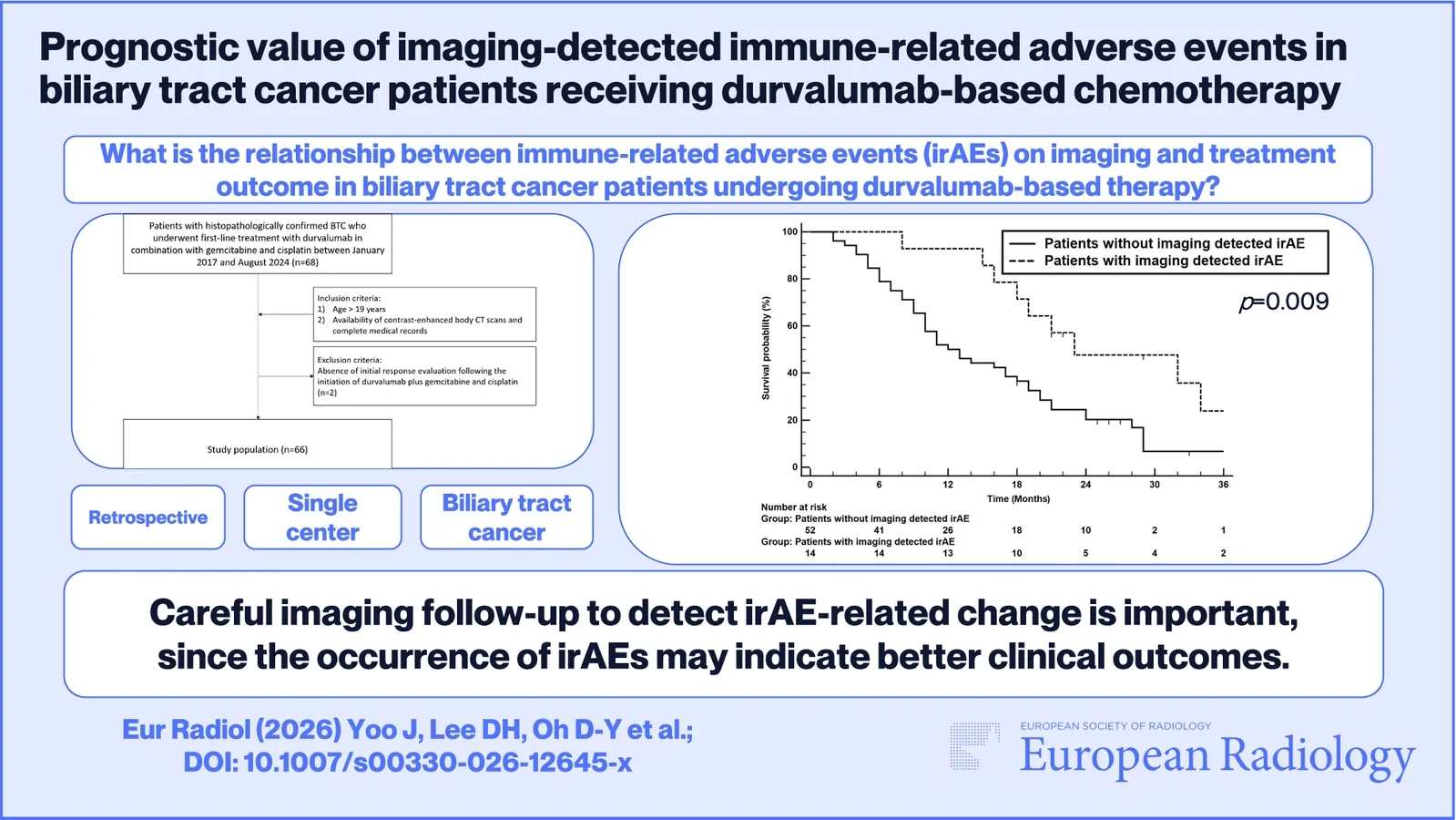

## Introduction

Biliary tract cancer (BTC), a heterogeneous group of malignancies encompassing intrahepatic and extrahepatic cholangiocarcinoma and gallbladder cancer, is a lethal disease with reported 5-year survival rates of 28.5%, 19.9%, and 10.8%, respectively [1]. Surgical resection is considered the only potentially curative treatment for BTC, but most patients with BTC have advanced cancer that is not amenable to surgical resection [2]. In patients with advanced or metastatic stage, systemic chemotherapy represents the standard of care [3].

Recently, immunotherapy has emerged as a promising treatment modality for various cancers, including BTC, with immune checkpoint inhibitors (ICI) such as durvalumab or pembrolizumab showing potential in combination with conventional chemotherapy regimens [4]. Durvalumab, an anti-programmed death ligand-1 (PD-L1) monoclonal antibody, added to first-line chemotherapy with gemcitabine and cisplatin, has been shown to improve overall survival (OS) in the TOPAZ-1 trial [4]. Furthermore, pembrolizumab, an anti-PD-1 immune checkpoint inhibitor, significantly improved OS when combined with first-line chemotherapy, compared to placebo in the KEYNOTE-966 trial [5]. Based on these results, the combination therapy of durvalumab or pembrolizumab, gemcitabine, and cisplatin has been recommended as the preferred regimen for advanced BTC by National Comprehensive Cancer Network (NCCN) guidelines [6].

Unlike conventional cytotoxic chemotherapy, ICIs are associated with distinct toxicities termed immune-related adverse events (irAEs). These events typically manifest within the first few months of treatment initiation [7]. Although the pathophysiology of irAEs remains to be fully understood, it is presumed that, in predisposed patients or in the presence of pre-existing conditions, ICIs may overstimulate the immune system and alter host homeostasis, causing an excessive inflammatory response. A broad range of irAEs involve almost every organ but mostly affect the skin, digestive system, lung, endocrine glands, nervous system, kidney, blood cells, and musculoskeletal system [8]. Diagnostic imaging, specifically computed tomography (CT) and magnetic resonance imaging (MRI), is able to detect and characterize irAEs [9]. Existing evidence suggests that the occurrence of certain irAEs may correlate with favorable clinical outcomes, such as improved survival rates [10]. However, the relationship between imaging-detected irAEs and therapeutic outcomes in patients with BTC receiving combination therapy with ICI and conventional cytotoxic chemotherapy has not been thoroughly investigated.

Therefore, this study aims to evaluate the potential role of irAEs detected on imaging studies in predicting prognosis in BTC patients treated with a combination of durvalumab plus gemcitabine and cisplatin.

## Materials and methods

This retrospective observational cohort study was approved by the institutional review board (approval number: 2411-095-1587). The requirement for written informed consent was waived due to the retrospective nature of the study. All procedures were conducted in accordance with the ethical principles outlined in the Declaration of Helsinki of the World Medical Association.

### Patients

**Fig. 1:**
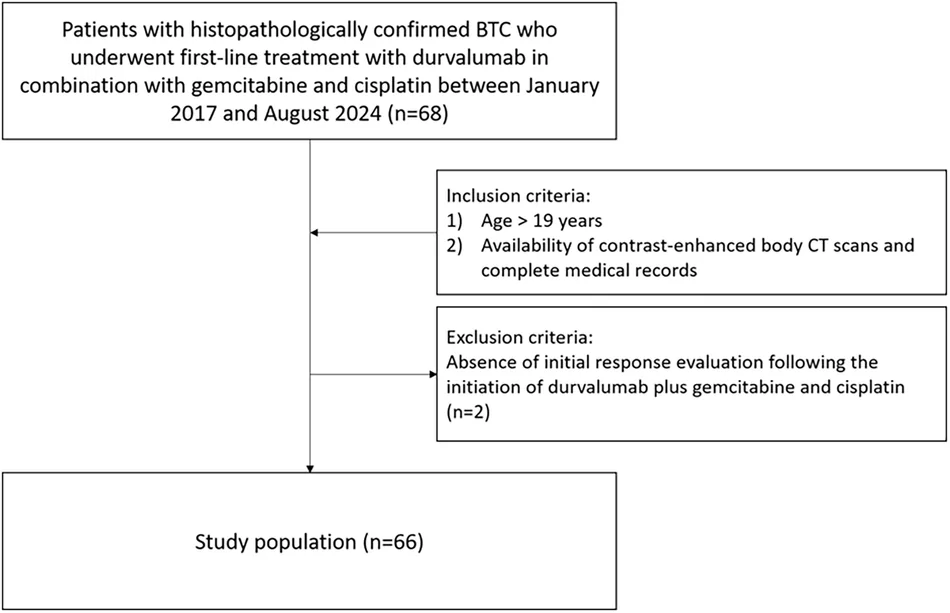
Patient enrollment process. BTC, biliary tract cancer

The inclusion criteria were as follows: (1) patients aged over 19 years; (2) histopathologically confirmed diagnosis of BTC; (3) receipt of first-line treatment with durvalumab in combination with gemcitabine and cisplatin for unresectable or recurrent BTC at our institution between January 2017 and August 2024; and (4) availability of contrast-enhanced body CT scans and complete medical records (Fig. 1). The exclusion criteria was the absence of initial response evaluation following the initiation of durvalumab plus gemcitabine and cisplatin.

### Treatment protocol

Durvalumab combined with gemcitabine and cisplatin was administered intravenously on a 21-day cycle [11]. Durvalumab (1500 mg) with or without tremelimumab (75 mg) was administered on day 1 of each cycle, in combination with gemcitabine (1000 mg/m^2^) and cisplatin (25 mg/m^2^), which were administered on days 1 and 8 of each cycle [11]. Treatment was continued until clinical or imaging (per Response Evaluation Criteria in Solid Tumors (RECIST) v1.1) disease progression or until unacceptable toxicity, or patient refusal.

### Assessment of outcome

All adverse events (AEs), including immune-related adverse events (irAEs), were assessed based on the National Cancer Institute’s Common Terminology Criteria for Adverse Events (CTCAE), version 5.0. This grading system categorizes severity from grade 1 to grade 5, with grade 5 indicating death due to an AE, and grade 0 indicating no symptoms or abnormalities. irAEs were defined as events with a suspected immunologic mechanism that required close monitoring and, when necessary, intervention with immunosuppressive agents such as corticosteroids or hormone replacement therapy [12]. AEs occurring more than 30 days after the final administration of durvalumab were excluded from analysis [13]. In addition, to identify any imaging findings suggestive of irAE, all imaging studies, including contrast-enhanced multiphasic abdominopelvic computed tomography (CT) and chest CT scans, were independently reviewed by two board-certified radiologists (16 and 8 years’ experience in body imaging interpretation, respectively), and discrepancies were resolved through a consensus discussion. As certain imaging features of irAEs, such as pulmonary infiltrates, may resemble those caused by other inflammatory conditions like infections, clinical symptoms and laboratory data were also considered to determine the occurrence of irAE-related imaging manifestations [14–16]. Detailed CT image acquisition parameters are described in the Supplementary Material.

### Endpoints

The primary endpoint was OS, and the secondary endpoint was progression-free survival (PFS), treatment response, and the incidence of irAEs during treatment. OS was calculated as the interval between the first day of durvalumab plus gemcitabine and cisplatin and death or the last follow-up date. The survival data of the study population were acquired from the national registry data from the Korean Ministry of Interior and Safety. The data cut-off date was December 1, 2024. PFS was defined as the time from the initiation of durvalumab plus gemcitabine and cisplatin until the date of RECIST version 1.1–defined imaging disease progression or death.

### Statistical analysis

All statistical analyses were performed using IBM SPSS Statistics for Windows version 29.0 (IBM Corp.) and MedCalc Statistical Software version 18.9.1 (MedCalc Software bvba; https://www.medcalc.org; 2019). Normality of continuous variables was assessed using the Shapiro–Wilk test. Categorical variables were presented as frequencies (%), and normally distributed continuous variables and non-normally distributed continuous variables were presented as means with standard deviation and medians with interquartile ranges (IQRs), respectively. Categorical variables were compared using the Chi-square test, whereas continuous variables were compared using the Mann–Whitney test. Univariate and multivariate Cox proportional hazards logistic regression analyses were performed to identify significant predictors of each outcome. All variables with *p*-values less than 0.05, in univariate analyses, were included in the multivariate analysis using stepwise selection. The Kaplan–Meier method was used for the estimation of the cumulative incidence of each outcome. Interobserver agreement between the reviewers was assessed using linear-weighted Kappa statistics. The interpretation of kappa values was as follows: < 0.20, poor agreement; 0.21–0.40, fair; 0.41–0.60, moderate; 0.61–0.80, substantial; and 0.81–1.00, almost perfect agreement. A *p*-value < 0.05 indicated statistical significance.

## Results

### Patient characteristics

Initially, 68 patients who were diagnosed as having BTC confirmed by histopathology and treated with durvalumab plus gemcitabine and cisplatin were identified. Among them, two patients were excluded from the analysis due to the absence of response evaluation after the start of treatment. The final cohort included 66 patients (mean age ± standard deviation, 64.7 ± 9.4 years; range, 35–85), of whom 35 (53.0%) were female. Primary tumor types included intrahepatic cholangiocarcinoma (*n* = 39, 59.1%), perihilar cholangiocarcinoma (*n* = 6, 9.1%), distal cholangiocarcinoma (*n* = 4, 6.1%), and gallbladder cancer (*n* = 17, 25.8%). In 15 patients (22.7%), tremelimumab was additionally administered. At diagnosis, disease status was either initially unresectable (*n* = 36, 54.5%) or recurrent following surgical resection (*n* = 30, 45.5%).

### Development of imaging-detected irAE

Imaging manifestations of irAEs were identified in 14 patients (21.2%) (Table 1). The most prevalent finding was bowel wall thickening (*n* = 5), followed by transient inflammatory lung nodules (*n* = 4). Among the 14 patients with imaging-detected irAEs, five also presented with clinical symptoms related to irAE. Detailed clinical manifestations and their correlation with imaging findings are provided in Supplementary Tables S1 and S2. Statistical analysis revealed no significant differences between patients with imaging-detected irAEs (*n* = 14) and those without (*n* = 52) regarding age, sex, primary tumor type, disease status, surgical history, or chemotherapy regimen (all *p* > 0.05) (Table 2). The median time to irAE onset was 56 days (interquartile range (IQR), 39–83 days), with 85.7% (12/14) of events occurring within the first 90 days following treatment initiation.

**Table 1:** Imaging-detected irAEs in 66 patients who underwent durvalumab plus gemcitabine and cisplatin with or without tremelimumab

	No. of patients (%)
Bowel wall thickening	5 (7.6%)
Transient inflammatory lung nodules	4 (6.1%)
Focal ground glass opacity	2 (3.0%)
Subcutaneous infiltration of the abdominal wall (panniculitis)	2 (3.0%)
Mesenteric panniculitis	2 (3.0%)
Axillary lymphadenitis	1 (1.5%)
Any	14* (21.2%)

*irAE* immune-related adverse event

* Two patients had more than one radiological irAE (axillary lymphadenitis and mesenteric panniculitis; inflammatory lung nodules and mesenteric panniculitis)

**Table 2:** Patient demographics and baseline characteristics according to the occurrence of immune-related adverse events

Characteristics	With imaging-detected irAE (*n* = 14)	Without imaging-detected irAE (*n* = 52)	*p*-value
Age (years, mean ± SD)	64.4 ± 6.1	64.8 ± 10.1	0.754
Sex			0.730
Male	6 (42.9%)	25 (48.1%)	
Female	8 (57.1%)	27 (51.9%)	
Primary tumor type			0.341
Cholangiocarcinoma	9 (64.3%)	40 (76.9%)	
Gallbladder	5 (35.7%)	12 (23.1%)	
Disease status			0.703
Initially unresectable	7 (50.0%)	29 (55.8%)	
Recurrent	7 (50.0%)	23 (44.2%)	
Virology status			0.288
No viral hepatitis	14 (100.0%)	48 (92.3%)	
Any viral hepatitis B	0	4 (7.7%)	
Active viral hepatitis B	0	3 (5.8%)	
Prior hepatitis C	0	0	
Initial serum CA 19-9 level (U/mL, median [IQR])	331.5 (24.0–1489.0)	49.7 (14.0–2180.0)	0.621
Surgical resection			0.800
Yes	7 (50.0%)	24 (46.2%)	
No*	7 (50.0%)	28 (53.8%)	
Chemotherapy protocol			0.560
Durvalumab plus gemcitabine and cisplatin	10 (71.4%)	41 (78.8%)	
Durvalumab plus gemcitabine and cisplatin with tremelimumab	4 (28.6%)	11 (21.2%)	
Distant metastasis at the initiation of durvalumab-based therapy			0.341
Presence	9 (64.3%)	40 (76.9%)	
Absence	5 (35.7%)	12 (23.1%)	
Biliary drainage at the initiation of durvalumab-based therapy			0.903
Yes	4 (28.6%)	14 (26.9%)	
No	10 (71.4%)	38 (73.1%)	

*irAE* immune-related adverse event, *SD* standard deviation, *IQR* interquartile range, *CA 19-9* carbohydrate antigen 19-9

* One out of 36 patients with initially unresectable tumors underwent palliative cholecystectomy

### Predictors for OS

**Fig. 2:**
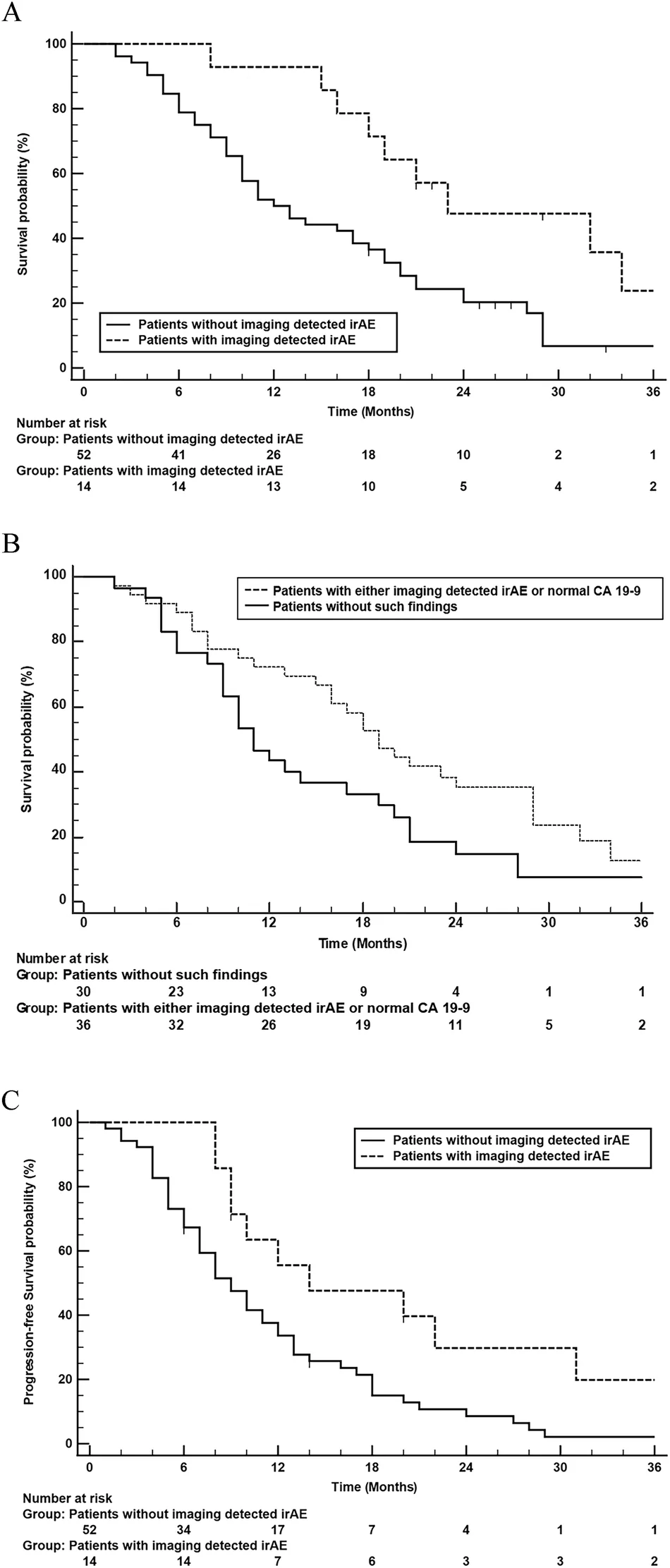
Kaplan–Meier estimates according to the development of immune-related adverse events. **A**, **B** Overall survival; **C** progression-free survival. irAE, immune-related adverse event

During the median follow-up period of 16.5 months (interquartile range (IQR), 9.0–24.0 months; range, 2–81), 56 (84.8%) patients died. Causes of deaths were tumor progression (*n* = 26, 46.4%), sepsis due to cholangitis (*n* = 15, 26.7%), unknown (*n* = 5, 8.9%), gastrointestinal bleeding (*n* = 3, 5.4%), pneumonia (*n* = 3, 5.4%), hepatic failure (*n* = 2, 3.6%), pulmonary thromboembolism (*n* = 1, 1.8%), and cerebral infarction (*n* = 1, 1.8%). The estimated 1-, 2-, and 3-year OS rates for the entire cohort were 59.1%, 26.2%, and 10.4%, respectively. The occurrence of irAEs, identified radiologically (hazard ratio (HR) = 0.41, *p* = 0.013), and baseline serum CA 19-9 level (HR = 1.01, *p* = 0.009) were significantly associated with OS in univariate analyses (Table 3). In multivariate analysis, the presence of imaging-detected irAEs (HR = 0.42; 95% confidence interval (CI), 0.21–0.86; *p* = 0.017) remained an independent predictor of OS, together with serum CA 19-9 level. The median OS after initiation of durvalumab-based chemotherapy was 23.0 months in patients who developed irAEs detected on imaging study (*n* = 14) and 12.0 months in those without (*n* = 52). Furthermore, the estimated OS rates at 1, 2, and 3 years were significantly higher in the imaging-detected irAE group (92.9%, 47.6%, and 23.8%, respectively) than in the group without such findings (50.0%, 20.3%, and 0.0%, respectively; *p* = 0.009) (Fig. 2A). Harrell’s C-index of a prediction model using imaging-detected irAEs was 0.591 (95% CI, 0.545–0.637) and that of a model using both imaging-detected irAE and baseline serum CA 19-9 level was 0.642 (95% CI, 0.570–0.713). The estimated OS rates at 1, 2, and 3 years were significantly higher in the subgroup with either imaging-detected irAEs or baseline CA 19-9 < 37 U/mL (72.2%, 35.3%, and 12.5%, respectively) than in the subgroup without such findings (43.3%, 14.8%, and 7.4%, respectively; *p* = 0.038) (Fig. 2B).

**Table 3:** Cox regression analyses for predicting overall survival in 66 patients with biliary tract cancer

Characteristics	Univariate analysis		Multivariate analysis	
	Hazard ratio (95% CI)	*p*-value	Hazard ratio (95% CI)	*p*-value
Age (years)	1.02 (0.98–1.05)	0.362		
Sex (male versus female)	1.61 (0.94–2.77)	0.083		
Primary tumor (cholangiocarcinoma vs. gallbladder cancer)	0.97 (0.52–1.80)	0.920		
Initial serum CA 19-9 level (U/mL)	1.01 (1.01–1.01)	0.009	1.01 (1.01–1.01)	**0.013**
Presence of imaging-detected irAE*	0.41 (0.20–0.83)	0.013	0.42 (0.21–0.86)	**0.017**
Presence of symptomatic irAE	0.84 (0.44–1.59)	0.595		
Presence of distant metastasis at the initiation of therapy	1.85 (0.95–3.60)	0.070		
Performance of biliary drainage at the initiation of therapy	1.16 (0.63–2.14)	0.638		

*CI* confidence interval, *CA 19-9* carbohydrate antigen 19-9, *irAE* immune-related adverse event

Bold values denote statistical significance at the *p* < 0.05 level

### Predictors for PFS

During the follow-up period, the estimated 1-, 2-, and 3-year PFS rates were 38.2%, 13.0%, and 5.6%, respectively. Presence of irAE detected on imaging study (HR = 0.43, *p* = 0.016) was the only significant predictor of PFS (Supplementary Table S3). Patients with imaging-detected irAEs demonstrated significantly higher estimated 1-, 2-, and 3-year PFS rates (55.6%, 29.8%, and 19.8%, respectively) compared to those without such findings (33.7%, 8.6%, and 0.0%, respectively; *p* = 0.011) (Fig. 2C).

### Treatment response

**Fig. 3:**
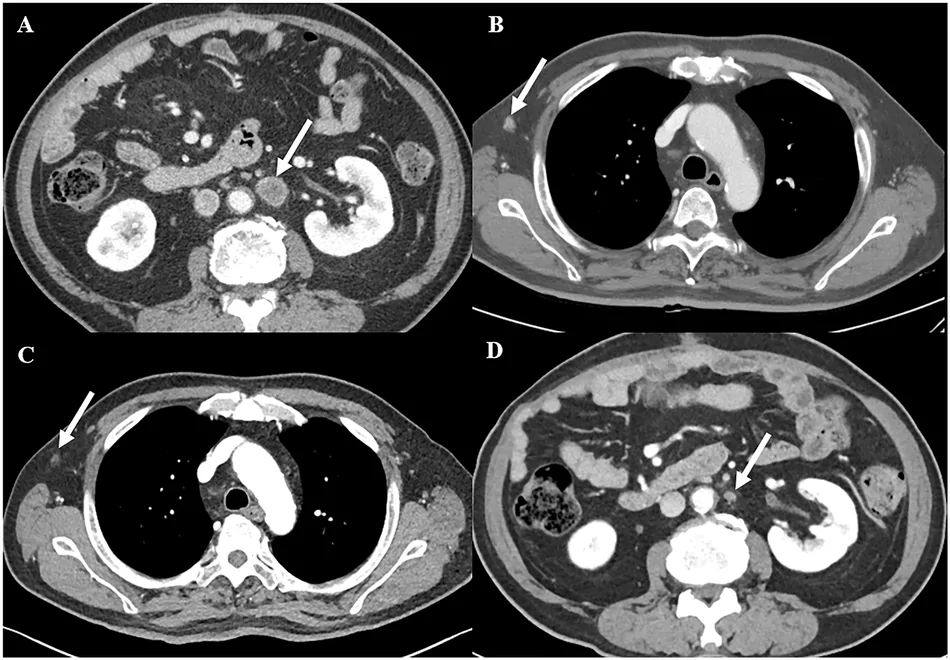
A 62-year-old man with recurrent intrahepatic cholangiocarcinoma following surgery. **A** Contrast-enhanced axial portal venous phase CT obtained 18 months after right hemihepatectomy demonstrates a 2-cm enlarged lymph node in the left para-aortic region (arrow), consistent with retroperitoneal lymph node metastasis. The patient subsequently received treatment with durvalumab, gemcitabine, cisplatin, and tremelimumab. **B** Follow-up contrast-enhanced axial chest CT at 2 months after initiating treatment including durvalumab reveals mild enlargement of a right axillary lymph node (arrow). **C** Follow-up contrast-enhanced axial chest CT at 4 months demonstrates a spontaneous decrease in the size of the previously enlarged right axillary lymph node (arrow). **D** Concurrent contrast-enhanced axial portal venous phase CT at 4 months shows a marked reduction in the size of the metastatic left para-aortic lymph node (arrow), consistent with a partial response to treatment. The patient ultimately died of disease progression 34 months after initiation of durvalumab-based therapy

Among the 14 patients with imaging-detected irAEs, overall responses included complete response (CR) in two patients, partial response (PR) in five, and stable disease (SD) in seven (Fig. 3 and Supplementary Table S4). Conversely, in the group without imaging-detected irAEs (*n* = 52), one patient achieved CR, 15 achieved PR, 32 maintained SD, and four experienced progressive disease (PD). There was no significant difference in objective response rate between patients with imaging-detected irAEs (50.0%) and those without (30.8%) (*p* = 0.183). Similarly, no significant difference in overall treatment response distribution was observed between the two groups (*p* = 0.157).

### Interobserver agreement regarding the presence of irAE on CT images

The weighted kappa was 0.637 (95% CI, 0.408–0.867) for the presence of irAE on CT images, indicating substantial interobserver agreement.

## Discussion

Following the success of the TOPAZ-1 and Keynote-966 trials, the combination of ICIs with gemcitabine and cisplatin has become the current standard first-line systemic therapy for unresectable BTC [4, 5]. With these milestone trials, recent NCCN and European Society for Medical Oncology guidelines also recommend durvalumab or pembrolizumab in combination with gemcitabine and cisplatin for this indication [3, 6]. Unlike traditional cytotoxic chemotherapies, ICIs are associated with a distinct spectrum of irAEs. A recent systematic review encompassing various cancers and ICIs reported a mean incidence rate of 40% for irAEs of any grade [17]. In addition, it has been reported that these adverse events commonly emerge within the initial 3 months after treatment initiation [18]. However, the specific incidence of imaging-detected irAEs in BTC patients treated with durvalumab plus gemcitabine and cisplatin has remained largely undocumented. In the present study, imaging-detected irAEs were observed in 21.2% (14/66) of patients. Furthermore, 84.0% of these events developed within 90 days of treatment initiation, a timeline consistent with previously reported data [18].

The immune system maintains homeostasis through the mechanism of self-tolerance to prevent autoimmunity. However, administration of ICIs may disrupt these regulatory pathways, resulting in excessive T-cell activation and proliferation. This activation subsequently stimulates B-cell responses and antibody production, ultimately leading to immune-mediated damage not only to tumor cells but also to normal tissues, manifesting as irAEs. Consequently, the occurrence of irAEs may reflect robust antitumor immune activity elicited by ICIs. Prior studies have shown that the presence of mild and manageable irAEs is associated with improved treatment outcomes [19, 20]. In our cohort, a substantial proportion of imaging-detected irAEs were subclinical; only five of 14 patients presented with clinical symptoms. All symptomatic cases were classified as Grade 1 or 2, reflecting a mild severity profile, and no patients required discontinuation of ICI therapy due to toxicity. These events were effectively managed with oral corticosteroids, hormone replacement, or supportive care. Furthermore, by evaluating follow-up imaging for features suggestive of irAEs, which may represent the earliest and most indolent forms of immune-related toxicity, we found that the presence of imaging-detected irAEs significantly correlated with prolonged median OS (23.0 months vs. 12.0 months; *p* = 0.009).

Previous studies have also demonstrated an association between irAEs and favorable clinical outcomes in other malignancies, including breast cancer and non-small-cell lung cancer treated with durvalumab [19, 21]. Although data regarding the prognostic implications of irAEs in patients with BTC receiving durvalumab-based chemotherapy remain limited, our findings align with these established trends. Notably, while earlier studies predominantly focused on clinical manifestations, the present study specifically evaluated imaging-detected irAEs. Given that certain toxicities, such as pneumonitis, may manifest radiologically before the onset of clinical symptoms [20], these findings highlight the necessity of careful imaging evaluation during follow-up to detect early irAE-related changes.

Considering the clinical impact of irAEs on treatment response and survival, efforts have been made to identify predictors of irAE development. However, due to the heterogeneity of disease mechanisms and therapeutic agents, current data remain limited [20, 22]. In our analysis, baseline characteristics did not differ significantly between patients who did and did not experience irAEs detected on imaging study, and there was no factor associated with the development of irAE after initiation of durvalumab-based chemotherapy for BTC patients.

This study has several limitations. Most notably, its retrospective design and relatively small sample size may introduce selection bias. Nonetheless, as durvalumab plus gemcitabine and cisplatin has only recently been established as a standard regimen for BTC patients following the TOPAZ-1 trial, patient recruitment remains challenging even in high-volume tertiary centers. Future research with larger cohorts is needed to validate our findings.

In summary, our study demonstrates that the occurrence of imaging-detected irAEs is significantly associated with improved overall survival in BTC patients undergoing durvalumab-based systemic therapy.

## Supplementary information

Supplementary information

## Abbreviations

AEs: Adverse events
BTC: Biliary tract cancer
CI: Confidence interval
CR: Complete response
CT: Computed tomography
CTCAE: National Cancer Institute’s Common Terminology Criteria for Adverse Events
ICI: Immune checkpoint inhibitors
IQRs: Interquartile ranges
irAE: Immune-related adverse events
NCCN: National Comprehensive Cancer Network
OS: Overall survival
PFS: Progression-free survival
PR: Partial response
RECIST: Response Evaluation Criteria in Solid Tumors
SD: Stable disease
