# Supplementary material for: Prognostic value of imaging-detected immune-related adverse events in biliary tract cancer patients receiving durvalumab-based chemotherapy
Source: Eur Radiol. 2026 May 28;36(10):8012–20. doi: 10.1007/s00330-026-12645-x (PMC13574883; doi:10.1007/s00330-026-12645-x)
Supplement: Supplementary file 1 [file 330_2026_12645_MOESM1_ESM.pdf]

ELECTRONIC SUPPLEMENTARY MATERIAL

Prognostic Value of Imaging-Detected Immune-Related Adverse Events in Biliary Tract Cancer Patients Receiving Durvalumab-Based Chemotherapy

STROBE Statement—Checklist of items that should be included in reports of ***cohort studies***

	Item No	Recommendation	Page No
**Title and abstract**	1	(*a*) Indicate the study's design with a commonly used term in the title or the abstract<br>(*b*) Provide in the abstract an informative and balanced summary of what was done and what was found	2
**Introduction**			
Background/rationale	2	Explain the scientific background and rationale for the investigation being reported	4-5
Objectives	3	State specific objectives, including any prespecified hypotheses	5
**Methods**			
Study design	4	Present key elements of study design early in the paper	6
Setting	5	Describe the setting, locations, and relevant dates, including periods of recruitment, exposure, follow-up, and data collection	6-7
Participants	6	(*a*) Give the eligibility criteria, and the sources and methods of selection of participants. Describe methods of follow-up<br>(*b*) For matched studies, give matching criteria and number of exposed and unexposed	6-7
Variables	7	Clearly define all outcomes, exposures, predictors, potential confounders, and effect modifiers. Give diagnostic criteria, if applicable	7
Data sources/ measurement	8*	For each variable of interest, give sources of data and details of methods of assessment (measurement). Describe comparability of assessment methods if there is more than one group	7
Bias	9	Describe any efforts to address potential sources of bias	NA
Study size	10	Explain how the study size was arrived at	7
Quantitative variables	11	Explain how quantitative variables were handled in the analyses. If applicable, describe which groupings were chosen and why	8
Statistical methods	12	(*a*) Describe all statistical methods, including those used to control for confounding<br>(*b*) Describe any methods used to examine subgroups and interactions<br>(*c*) Explain how missing data were addressed<br>(*d*) If applicable, explain how loss to follow-up was addressed<br>(*e*) Describe any sensitivity analyses	8
**Results**			
Participants	13*	(a) Report numbers of individuals at each stage of study—eg numbers potentially eligible, examined for eligibility, confirmed eligible, included in the study, completing follow-up, and analysed<br>(b) Give reasons for non-participation at each stage	9

		(c) Consider use of a flow diagram	
Descriptive data	14*	(a) Give characteristics of study participants (eg demographic, clinical, social) and information on exposures and potential confounders<br>(b) Indicate number of participants with missing data for each variable of interest<br>(c) Summarise follow-up time (eg, average and total amount)	9
Outcome data	15*	Report numbers of outcome events or summary measures over time	9-11

Main results	16	(*a*) Give unadjusted estimates and, if applicable, confounder-adjusted estimates and their precision (eg, 95% confidence interval). Make clear which confounders were adjusted for and why they were included<br>(*b*) Report category boundaries when continuous variables were categorized<br>(*c*) If relevant, consider translating estimates of relative risk into absolute risk for a meaningful time period	10-11
Other analyses	17	Report other analyses done—eg analyses of subgroups and interactions, and sensitivity analyses	11
**Discussion**			
Key results	18	Summarise key results with reference to study objectives	12
Limitations	19	Discuss limitations of the study, taking into account sources of potential bias or imprecision. Discuss both direction and magnitude of any potential bias	13-14
Interpretation	20	Give a cautious overall interpretation of results considering objectives, limitations, multiplicity of analyses, results from similar studies, and other relevant evidence	13-14
Generalisability	21	Discuss the generalisability (external validity) of the study results	14
**Other information**			
Funding	22	Give the source of funding and the role of the funders for the present study and, if applicable, for the original study on which the present article is based	NA

*Give information separately for exposed and unexposed groups.

Note: An Explanation and Elaboration article discusses each checklist item and gives methodological background and published examples of transparent reporting. The STROBE checklist is best used in conjunction with this article (freely available on the Web sites of PLoS Medicine at http://www.plosmedicine.org/, Annals of Internal Medicine at http://www.annals.org/, and Epidemiology at http://www.epidem.com/). Information on the STROBE Initiative is available at http://www.strobe-statement.org.

CT image acquisition

All patients underwent chest and abdominal CT scans, using various types of CT scanners owing to the retrospective study design. CT scanning was performed using the following parameters: tube voltage, 90–120 kVp according to the scanner type; tube current-time products, 100–300 mAs; rotation time, 0.5 s; pitch, 0.6–1.2; and slice thickness, 2.5–3 mm. The iobitridol (Xenetix 350; Guerbet) was intravenously injected at a dose of 520 mg/kg body weight using a power injector (Stellent D; Medrad) for 30 s at a rate of 2–5 mL/s according to body weight, followed by a 20–30-mL saline flush. The timing for chest and abdominal late arterial phase (LAP) and portal venous phase (PVP) scans was determined using the bolus tracking technique; the abdominal LAP scan was automatically performed 17 s after the attenuation coefficient of the abdominal aortic blood reached 100 HU. Chest CT and abdominal PVP images were acquired 50 s and 70 s, respectively, after the initiation of the contrast media administration.

Supplementary Table S1. Clinical manifestation of irAE in 66 patients who underwent durvalumab plus gemcitabine and cisplatin with or without tremelimumab

	**No. of patients (%)**	**Grade 1[†]**	**Grade 2[†]**
Skin			
Rash	5 (7.6%)	2	3
Pruritus	4 (6.1%)	4	0
Endocrine			
Hypothyroidism	3 (4.5%)	1	2
Adrenal insufficiency	1 (1.5%)	0	1
Immune thrombocytopenic purpura	2 (3.0%)	0	2
Enteritis	2 (3.0%)	0	2
Myositis	1 (1.5%)	0	1
Any	16* (24.2%)	7	11

Note. – irAE = immune-related adverse event; GGO = ground glass opacity; N/A = not applicable.
[†]Adverse events evaluated according to the National Cancer Institute Common Terminology Criteria for Adverse Events version 5.0
*Two patients had more than one clinical irAEs (pruritus and thyroiditis; pruritus and adrenal insufficiency).

Supplementary Table S2. Frequency of radiological and clinical irAE in patients with biliary tract cancer after treatment with durvalumab plus Gemcitabine and Cisplatin

	**Radiological irAE (+)**	**Radiological irAE (-)**	**Total**
**Clinical irAE (+)**	5 (7.6%)	11 (16.7%)	16 (24.2%)
**Clinical irAE (-)**	9 (13.6%)	41 (62.1%)	50 (75.8%)
**Total**	14 (21.2%)	52 (78.8%)	66 (100.0%)

Note. – irAE = immune-related adverse event

Supplementary Table S3. Cox regression analyses for predicting progression-free survival in 66 patients with biliary tract cancer

**Characteristics**	**Univariate analysis**		**Multivariate analysis**	
	Hazard ratio (95% CI)	*p*-value	Hazard ratio (95% CI)	*p*-value
**Age (y)**	1.03 (1.00–1.06)	0.085		
**Sex (male versus female)**	1.15 (0.69–1.92)	0.598		
**Primary tumor location (cholangiocarcinoma versus gallbladder cancer)**	0.78 (0.43–1.43)	0.423		
**Initial serum CA 19-9 level (U/mL)**	1.00 (1.00–1.00)	0.120		
**Presence of imaging-detected irAE**	0.43 (0.22-0.85)	**0.016**	0.43 (0.22-0.85)	**0.011**
**Presence of distant metastasis at the initiation of durvalumab plus gemcitabine and cisplatin with/without tremelimumab**	1.56 (0.86–2.86)	0.146		
**Performance of biliary drainage at the initiation of durvalumab plus gemcitabine and cisplatin with/without tremelimumab**	1.27 (0.72–2.25)	0.523		

Note. – CI = confidence interval; CA 19-9 = carbohydrate antigen 19-9; irAE = immune-related adverse event

Supplementary Table S4. Comparison of treatment responses between patients with and without irAE

Characteristics	With imaging detected irAE (n=14)	Without imaging detected irAE (n=52)	*p*-value
**Best objective response**			0.157
Complete response	2 (14.3%)	1 (1.9%)	
Partial response	5 (35.7%)	15 (28.9%)	
Stable disease	7 (50.0%)	32 (61.5%)	
Progressive disease	0 (0.0%)	4 (7.7%)	
**Objective response rate**	7 (50.0%)	16 (30.8%)	0.183

Note. – irAE = immune-related adverse event
